# Associations of viral ribonucleic acid (RNA) shedding patterns with clinical illness and immune responses in Severe Acute Respiratory Syndrome Coronavirus 2 (SARS‐CoV‐2) infection

**DOI:** 10.1002/cti2.1160

**Published:** 2020-07-27

**Authors:** Pei Hua Lee, Woo Chiao Tay, Stephanie Sutjipto, Siew‐Wai Fong, Sean Wei Xiang Ong, Wycliff Enli Wei, Yi‐Hao Chan, Li Min Ling, Barnaby E Young, Matthias Paul HS Toh, Laurent Renia, Lisa FP Ng, Yee‐Sin Leo, David C Lye, Tau Hong Lee

**Affiliations:** ^1^ National Centre for Infectious Diseases Singapore; ^2^ Tan Tock Seng Hospital Singapore; ^3^ Singapore Immunology Network Agency for Science, Technology and Research Singapore; ^4^ Department of Biological Sciences National University of Singapore Singapore; ^5^ Lee Kong Chian School of Medicine Singapore; ^6^ Saw Swee Hock School of Public Health Singapore; ^7^ Institute of Infection, Veterinary and Ecological Sciences University of Liverpool Liverpool UK; ^8^ Yong Loo Lin School of Medicine Singapore

**Keywords:** COVID‐19, cytokines, immune responses, SARS‐CoV‐2, viral RNA shedding

## Abstract

**Objectives:**

A wide range of duration of viral RNA shedding in patients infected with Severe Acute Respiratory Syndrome Coronavirus 2 (SARS‐CoV‐2) has been observed. We aimed to investigate factors associated with prolonged and intermittent viral RNA shedding in a retrospective cohort of symptomatic COVID‐19 patients.

**Methods:**

Demographic, clinical and laboratory data from hospitalised COVID‐19 patients from a single centre with two consecutive negative respiratory reverse transcription‐polymerase chain reaction (RT‐PCR) results were extracted from electronic medical records. Kaplan–Meier survival curve analysis was used to assess the effect of clinical characteristics on the duration and pattern of shedding. Plasma levels of immune mediators were measured using Luminex multiplex microbead‐based immunoassay.

**Results:**

There were 201 symptomatic patients included. Median age was 49 years (interquartile range 16–61), and 52.2% were male. Median RNA shedding was 14 days (IQR 9–18). Intermittent shedding was observed in 77 (38.3%). We did not identify any factor associated with prolonged or intermittent viral RNA shedding. Duration of shedding was inversely correlated with plasma levels of T‐cell cytokines IL‐1β and IL‐17A at the initial phase of infection, and patients had lower levels of pro‐inflammatory cytokines during intermittent shedding.

**Conclusions:**

Less active T‐cell responses at the initial phase of infection were associated with prolonged viral RNA shedding, suggesting that early immune responses are beneficial to control viral load and prevent viral RNA shedding. Intermittent shedding is common and may explain re‐detection of viral RNA in recovered patients.

## Graphical abstract

We studied 201 patients with PCR‐confirmed COVID‐19 infection. We found median RNA shedding was 14 days and intermittent RNA shedding was observed in 38.3%. The only associated clinical factor with prolonged RNA shedding was invasive mechanical ventilation. Importantly, we observed in a subset of patients with cytokine analysis, that prolonged RNA shedding was associated with EGF, FGF‐2, GRO‐α and RANTES at the initial phase of infection. Intermittent RNA shedding was associated with lower levels of pro‐inflammatory cytokines.

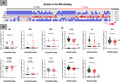

## Introduction

The global tally for coronavirus disease 2019 (COVID‐19) had crossed the 4.5‐millon mark and accounted for more than 300 000 deaths by 17 May 2000.^1^ The impact of the pandemic has placed an extra burden on healthcare systems, causing capacity limitations.^2^ In Singapore, all individuals with confirmed SARS‐CoV‐2 infection were initially admitted into airborne infection isolation rooms and attending staff wore personal protective equipment in accordance with the United States Centers for Disease Control and Prevention guidelines.^3^ More than 25 000 COVID‐19 cases have been reported in Singapore as of 16 May 2020.^4^ Of these, approximately 1095 (4.3%) were still hospitalised while 17 881 (71%) who were clinically well but still tested positive for severe acute respiratory syndrome coronavirus 2 (SARS‐CoV‐2), the virus causing COVID‐19, had been discharged to community isolation facilities.

A large proportion of patients with COVID‐19 experience only mild disease and do not require inpatient hospital care.^5^ However, several countries such as Singapore have a policy to hospitalise or isolate all patients with confirmed infection to mitigate the risk of secondary community transmission. However, the infectious period of COVID‐19 infection is at present still unclear, and the recommended duration of isolation required has varied across regulatory authorities.^6^, ^7^, ^8^ In a cohort of 191 patients in China, the median duration of viral ribonucleic acid (RNA) shedding by RT‐PCR was 20 days.^9^ Therefore, the length of stay in healthcare or isolation facilities can be much longer than medically required.

In a study of 113 patients in China, risk factors associated with prolonged SARS‐CoV‐2 RNA shedding included male gender, delayed admission to hospital after illness onset and requirement for invasive mechanical ventilation.^10^ At our centre, the National Centre for Infectious Diseases (NCID), we observed patients with prolonged and intermittent viral RNA shedding of SARS‐CoV‐2 RNA by RT‐PCR which precluded discharge from our healthcare or isolation facilities. In this report, we describe the prevalence of prolonged and intermittent viral RNA shedding and investigated associated factors as well as correlation with host immune responses.

## Results

### Baseline characteristics and clinical outcomes

We identified 205 patients who were hospitalised with at least one positive RT‐PCR for SAR‐CoV2‐2 and discharge followed by two consecutive negative results. As four patients were asymptomatic, they were excluded from analysis. Of the remaining 201 patients, 105 (52.2%) were male. The median age was 49 years (Interquartile range [IQR] 16–61 years). The common comorbidities were hypertension (23.4%), hyperlipidaemia (23.4%) and diabetes mellitus (13.9%). Median duration from illness onset to hospital admission was 5 days (IQR, 3–8 days). One hundred and six patients (52.7%) had pneumonia on chest radiograph, 43 (21.4%) required supplemental oxygen for hypoxia (oxygen saturation <94%), and 10 (5.0%) needed invasive mechanical ventilation. Death occurred in two patients. The patient characteristics are summarised in Table 1.

**Table 1:** Demographic and clinical characteristics of symptomatic patients with confirmed SARS‐CoV‐2 infection

	*N* = 201	Duration of viral RNA shedding from symptom onset		*P*
		≤ 14 days (*n* = 110)	> 14 days (*n* = 91)	
Median age, years (IQR)	49 (16–61)	48 (35–60)	51 (37–61)	0.465
Male	105 (52.2%)	54 (49.1%)	51 (56.0%)	0.395
Median days of hospitalisation (IQR)	13 (9–17)	11 (8–13)	17 (12–20)	**<0.001**
Co‐morbidities				
Diabetes mellitus	28 (13.9%)	15 (13.6%)	13 (14.3%)	1.000
Hypertension	47 (23.4%)	26 (23.6%)	21 (23.1%)	1.000
Hyperlipidaemia	47 (23.4%)	22 (20.0%)	25 (27.5%)	0.243
Ischemic heart disease	10 (5.0%)	4 (3.6%)	6 (6.6%)	0.353
Chronic lung disease	9 (4.5%)	3 (2.7%)	6 (6.6%)	0.305
Chronic kidney disease	2 (1.0%)	2 (1.8%)	0 (0.0%)	0.502
Chronic liver disease	1 (0.5%)	1 (0.9%)	0 (0.0%)	1.000
Cancer	10 (5.0%)	7 (6.4%)	3 (3.3%)	0.517
Severity				
Prolonged fever^a^	51 (25.4%)	23 (20.9%)	28 (30.8%)	0.143
Pneumonia	106 (52.7%)	53 (48.2%)	53 (58.2%)	0.160
Required supplemental oxygen	43 (21.4%)	23 (20.9%)	20 (22.0%)	0.865
Invasive mechanical ventilation	10 (5.0%)	2 (1.8%)	8 (8.8%)	0.045
Treatment				
Antiviral agent use	39 (19.4%)	17 (15.5%)	22 (24.2%)	0.152
Lopinavir/ritonavir	29 (14.4%)	10 (9.1%)	19 (20.9%)	**0.026**
Interferon‐beta 1b	12 (6.0%)	5 (4.5%)	7 (7.7%)	0.384
Remdesivir	9 (4.5%)	7 (6.4%)	2 (2.2%)	0.188
Hydroxychloroquine	5 (2.5%)	2 (1.8%)	3 (3.3%)	0.660
Blood results				
Total white count (min) ×10^9^ L^−1^	4.50 (3.50–5.63)	4.50 (3.50–5.78)	4.40 (3.58–5.50)	0.810
Total white count (max) ×10^9^ L^−1^	6.10 (5.10–7.75)	6.00 (4.80–7.75)	6.20 (5.30–7.60)	0.456
Haemoglobin g dL^−1^	13.40 (12.40–14.6)	13.50 (12.30–14.50)	13.40 (12.48–14.70)	0.976
Platelet (min) ×10^9^ L^−1^	193 (155–250)	191.00 (154.00–259.50)	196.00 (157.00–240.00)	0.996
Lymphocytes (min) ×10^9^ L^−1^	1.10 (0.74–1.57)	0.96 (0.76–1.53)	1.10 (0.71–1.38)	0.922
Lymphocytes (max) ×10^9^ L^−1^	1.57 (1.22–1.91)	1.56 (1.13–1.86)	1.57 (1.29–1.94)	0.477
Neutrophils (min) ×10^9^ L^−1^	2.59 (1.97–3.48)	2.61 (1.97–3.65)	2.58 (1.94–3.33)	0.631
Neutrophils (max) ×10^9^ L^−1^	4.16 (3.22–5.86)	4.17 (3.16–5.95)	4.15 (3.34–5.85)	0.951
Creatinine (max) μmol L^−1^	73.00 (57.75–86.25)	71.00 (55.00–86.50)	76.00 (60.00–86.50)	0.220
ALT (max) U L^−1^	31.00 (19.50–62.50)	29.50 (20.00–69.50)	33.00 (19.00–57.00)	0.962
LDH (max) U L^−1^	507.50 (388.25–681.25)	510.50 (388.75–659.00)	494.50 (386.25–686.75)	0.967
CRP (max) mg L^−1^	17.40 (4.70–74.10)	16.80 (4.50–77.10)	19.55 (4.70–74.08)	0.805

Continuous variables reported as median (interquartile range); discrete variables reported as number (percentage). The chi‐square (χ^2^) test or Fisher's exact test was used for comparison of discrete variables. *P* < 0.05 was deeemed to be significant.

ALT, Alanine transaminase; CRP, C‐reactive protein; LDH, Lactate dehydrogenase; Max, Maximum; Min, Minimum.≥ 7 days.

### Viral RNA shedding patterns and association with clinical characteristics

**Figure 1:**
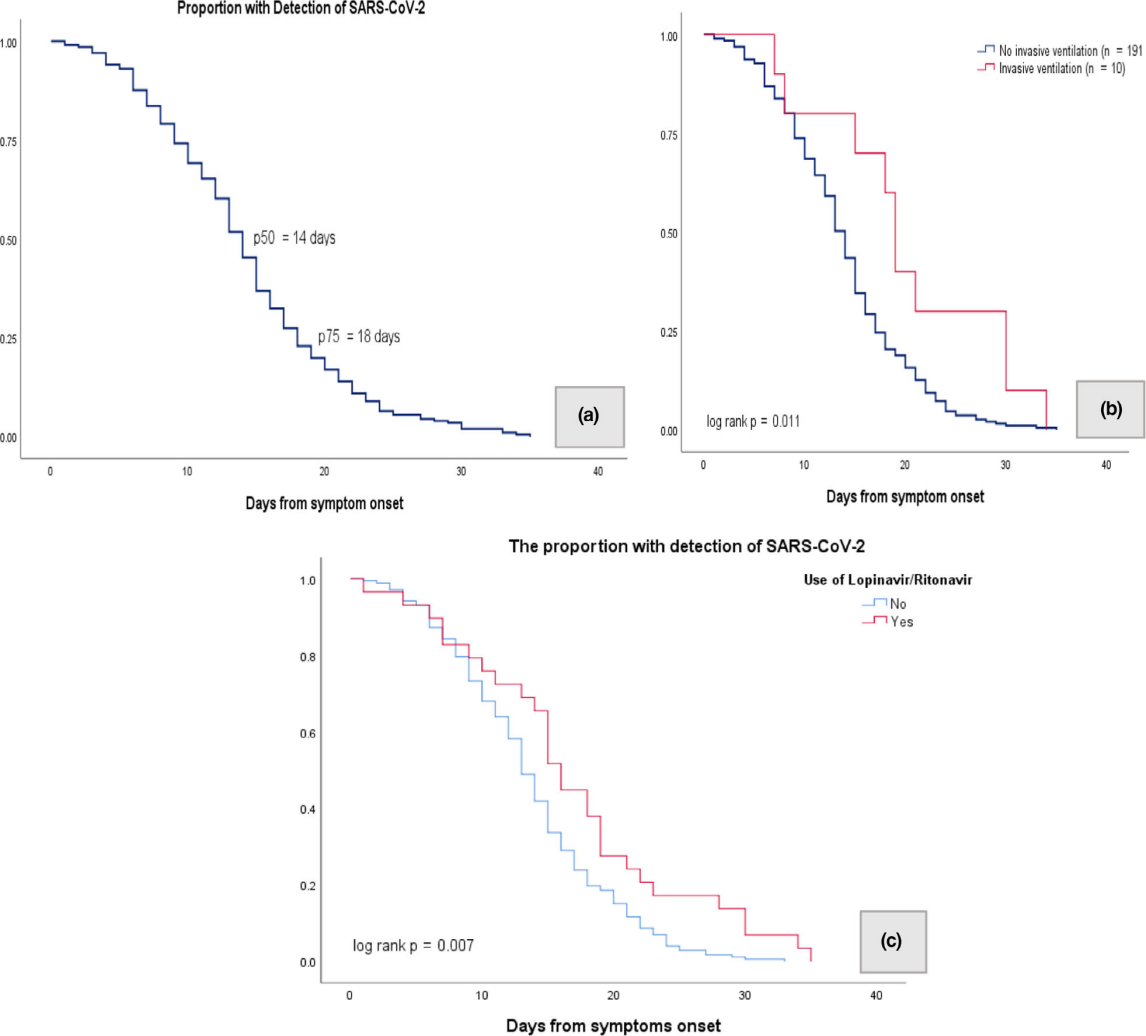
Kaplan–Meier curve. **(a)** The median duration of viral RNA shedding was 14 days. **(b)** The proportion of patients with detection of SARS‐CoV‐2 by days from symptom onset between patients who did not have invasive ventilation and patients who had invasive ventilation (log‐rank *P*‐value = 0.011). **(c)** The proportion of patients with detection of SARS‐CoV‐2 by days from symptom onset between patients who did not receive lopinavir/ritonavir treatment and patients who received lopinavir/ritonavir treatment (log‐rank *P*‐value = 0.007).

The median duration of viral RNA shedding in this study was 14 days (IQR 9–18) (Figure 1a). Prolonged viral shedding was thus defined as the duration of SARS‐CoV‐2 RNA shedding being longer than 14 days. The median duration of hospital stay was 13 days (IQR 9–17), and each patient received a median of 7 SARS‐CoV‐2 PCR tests. Duration of viral shedding was not significantly associated with sex, age, presence of comorbidities, baseline investigations, prolonged fever (≥ 7 days), pneumonia, need for supplemental oxygen and use of experimental antiviral agents. Median duration of viral RNA shedding was significantly longer in patients requiring invasive mechanical ventilation (19 days, 95% confidence interval [CI] 17.5–20.2) compared to 14 days (95% CI, 13.1–14.9) in patients not requiring mechanical ventilation, *P*‐value = 0.01 (Figure 1b). Duration of viral shedding was also significantly associated with lopinavir/ritonavir treatment (median 13 days, 95% CI 12.1–13.9) vs. 16 days (95% CI 14.3–17.8), *P*‐value = 0.026. However, in the multivariate logistic regression analysis, none of the factors analysed were statistically significant (Table 3).

**Figure 2:**
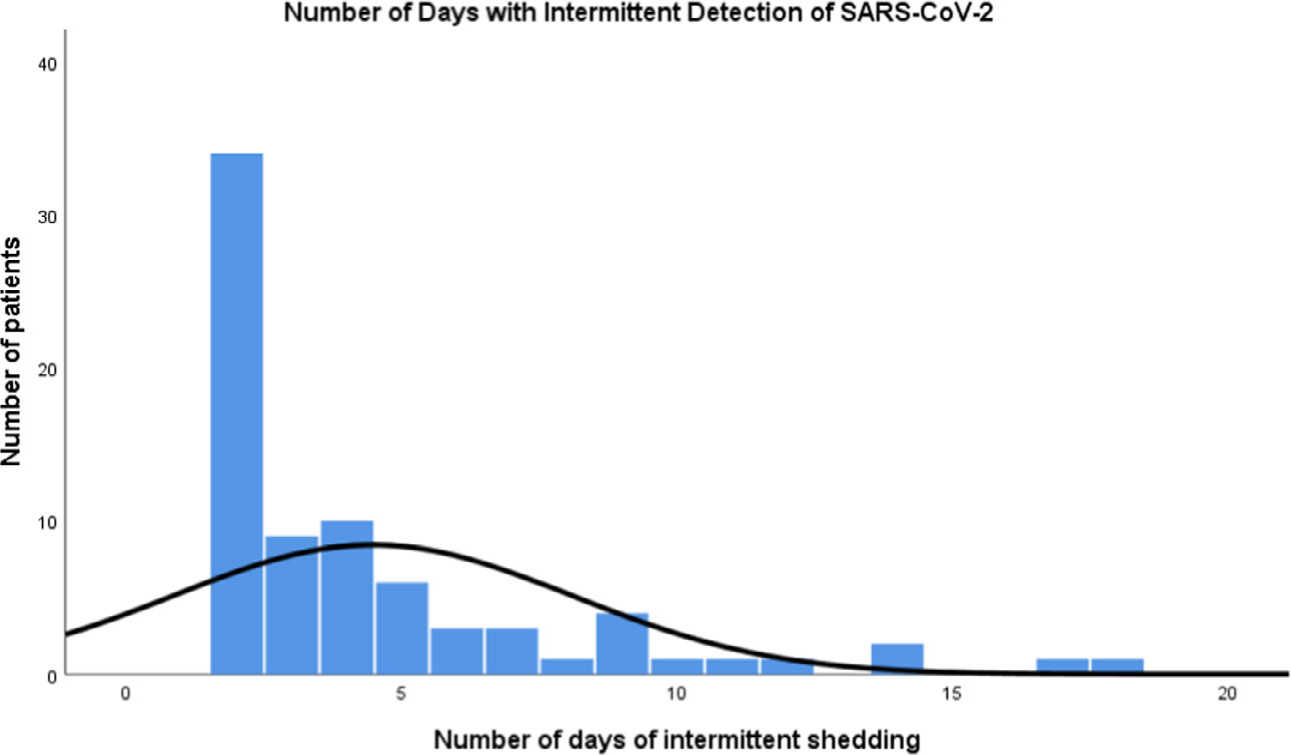
The median duration of intermittent viral RNA shedding was 3 days (*n* = 77).

Seventy‐seven patients (38.3%) had intermittent viral RNA shedding, which we defined as alternating between positive and negative PCR tests on serial testing, before cessation of viral shedding with two consecutive negative PCR tests. The median duration from symptom onset to intermittent viral RNA shedding was 13 days (IQR 10–16.5), and the median duration of intermittent viral RNA shedding was 3 days (IQR 2–5) (Figure 2). There was no significant gender difference in duration of viral RNA shedding among the 77 patients. There was also no significant difference in median duration of viral RNA shedding between different age groups, presence of comorbidities, prolonged fever (≥ 7 days), pneumonia, need for supplemental oxygen, use of experimental antiviral agents and invasive ventilation. Comparison of patients with intermittent viral RNA shedding (*n* = 77) to those without (*n* = 124) showed that the median duration of persistent viral RNA shedding before a negative RT‐PCR result was longer in patients with intermittent viral RNA shedding than patients without (13 days [95% CI 11.8–14.2] vs. 11 days [95% CI 9.6–12.4], *P*‐value <0.001). Length of hospital stay was 5 days longer in those with intermittent viral RNA shedding compared with those without (16 days [95% CI 14.6–17.4] vs. 11 days [95% CI 9.6–12.4], *P*‐value <0.001). There were no other significantly different clinical characteristics (Table 2).

**Table 2:** Demographic and clinical characteristics of patients with and without intermittent viral RNA shedding

	Patients *with* Intermittent Viral RNA Shedding (*n* = 77)	Patient *without* Non‐Intermittent Viral RNA Shedding (*n* = 124)	*P*
Median days of intermittent viral RNA shedding (IQR)	3 (2–5)	–	
Median days of persistent viral RNA shedding (before negative RT‐PCR result) (IQR)	13 (10–16.5)	11 (8–15)	**<0.001**
Median age, years (IQR)	48 (33–56)	51 (36–62)	0.854
Male	38 (49.4%)	67 (54.0%)	0.563
Median days of hospitalisation (IQR)	16 (12–19)	11 (8–15)	**<0.001**
Co‐morbidities			
Diabetes mellitus	10 (13.0%)	18 (14.5%)	0.836
Hypertension	16 (20.8%)	31 (25.0%)	0.607
Hyperlipidaemia	16 (20.8%)	31 (25.0%)	0.607
Ischemic heart disease	2 (2.6%)	8 (6.5%)	0.323
Chronic lung disease	5 (6.5%)	4 (3.2%)	0.308
Chronic kidney disease	0 (0.0%)	2 (1.6%)	0.525
Chronic liver disease	0 (0.0%)	1 (0.8%)	1.000
Cancer	1 (1.3%)	9 (7.3%)	0.092
Severity			
Prolonged fever ^a^	18 (23.4%)	33 (26.6%)	0.739
Pneumonia	40 (51.9%)	66 (53.2%)	0.885
Required supplemental oxygen	13 (16.9%)	30 (24.2%)	0.288
Invasive mechanical ventilation	5 (6.5%)	5 (4.0%)	0.511
Treatment			
Antiviral agents use	13 (16.9%)	26 (21.0%)	0.583
Lopinavir/ritonavir	11 (14.3%)	18 (14.5%)	1.000
Interferon‐beta	4 (5.2%)	8 (6.5%)	1.000
Remdesivir	1 (1.3%)	8 (6.5%)	0.157
Hydroxychloroquine	1 (1.3%)	4 (3.2%)	0.651
Blood results			
Total white count (min) ×10^9^ L^−1^	4.40 (3.60–5.30)	4.60 (3.40–5.80)	0.588
Total white count (max) ×10^9^ L^−1^	6.20 (5.20–7.28)	6.00 (4.90 – 8.40)	0.829
Haemoglobin g dL^−1^	13.20 (12.35–14.75)	13.50 (12.48–14.50)	0.765
Platelet (min) ×10^9^ L^−1^	185 (155.6–229)	196.50 (155.25–261.00)	0.264
Lymphocytes (min) ×10^9^ L^−1^	1.2 (0.76–1.39)	1.1 (0.70–1.51)	0.984
Lymphocytes (max) ×10^9^ L^−1^	1.63 (1.30–1.90)	1.49 (1.11–1.91)	0.260
Neutrophils (min) ×10^9^ L^−1^	2.58 (2.03–3.31)	2.61 (1.94–3.65)	0.809
Neutrophils (max) ×10^9^ L^−1^	4.09 (3.25–5.45)	4.25 (3.16–6.79)	0.408
Creatinine (max) μmol L^−1^	67.50 (58.00–84.00)	75.00 (57.00–88.00)	0.101
ALT (max) U L^−1^	25.00 (17.25–61.75)	34.00 (21.50–62.50)	0.122
LDH (max) U L^−1^	473.00 (391.00–646.00)	524.00 (388.00–718.00)	0.257
CRP (max) mg L^−1^	18.80 (5.00–64.80)	16.80 (4.15–108.35)	0.795

Continuous variables reported as median (interquartile range); discrete variables reported as number (percentage). Chi‐square (χ^2^) test or Fisher's exact test was used for comparison of discrete variables. *P* < 0.05was deemed to be significant.

ALT, Alanine transaminase; CRP, C‐reactive protein; LDH, Lactate dehydrogenase; Max, Maximum; Min, Minimum. ≥ 7 days.

### Viral RNA shedding and immune responses

**Figure 3:**
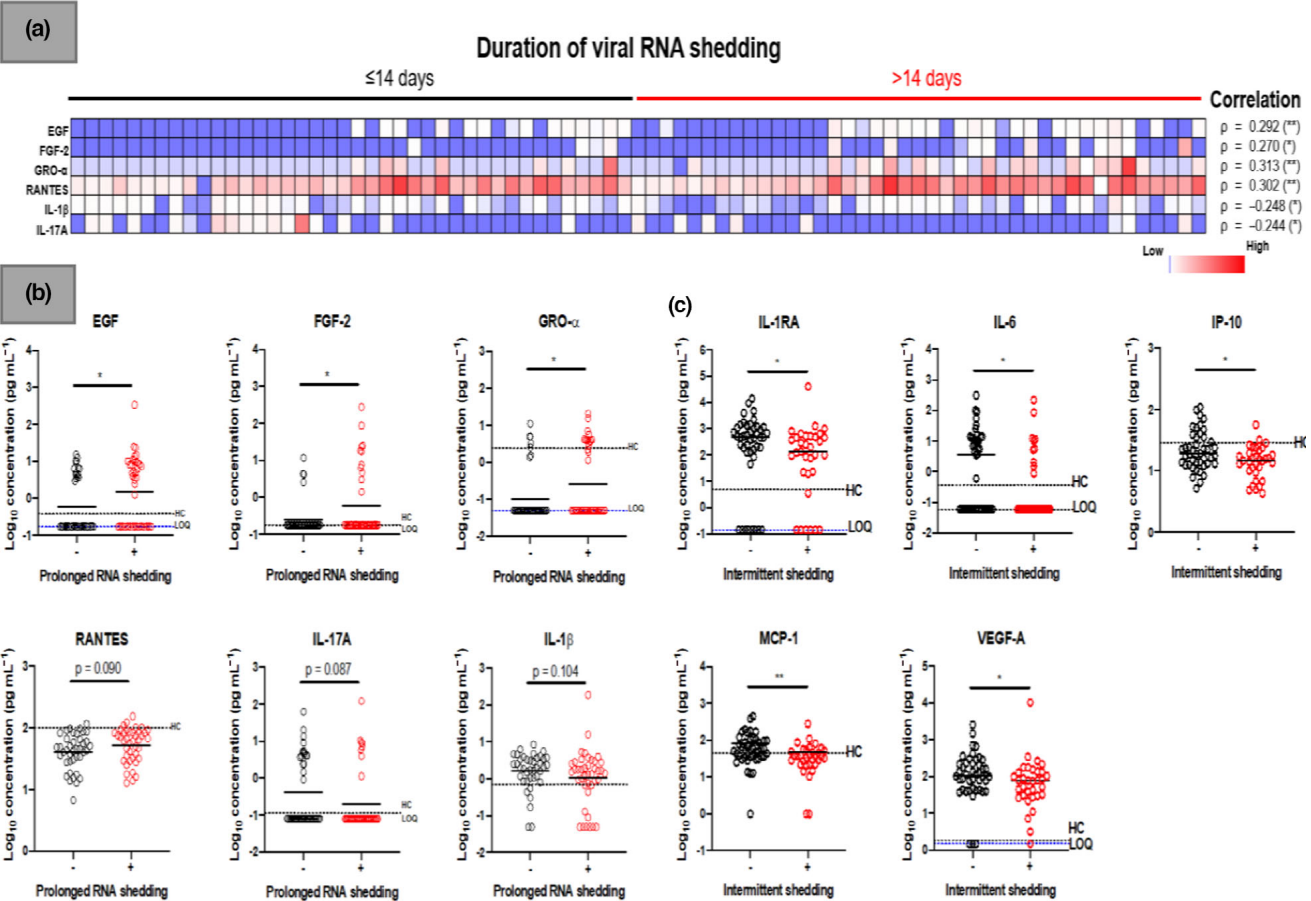
Association of plasma immune mediators levels and viral RNA shedding patterns in COVID‐19 patients. Concentrations of 45 immune mediators were quantified using a 45‐plex microbead‐based immunoassay. **(a)** Heatmap of significant immune mediators in patients with different duration of viral RNA shedding (≤ 14 days, *n* = 40; > 14 days, *n* = 41). Each colour represents the relative concentration of a particular analyte. Blue and red indicate low and high concentrations, respectively. Correlation of immune mediators at the acute phase (median 8 days PIO) with duration of viral RNA shedding in COVID‐19 patients. Spearman rank correlation analysis was conducted on plasma collected at the acute phase (median 8 days PIO) with duration of viral RNA shedding in COVID‐19 patients. Results of the correlation of immune mediators against duration of viral RNA shedding are presented as rho (ρ) and asterisks (**P‐value < 0.01, *P‐value < 0.05). **(b)** Profiles of immune mediators between patients with or without prolonged viral RNA shedding (> 14 days) and C, patients with or without intermittent viral RNA shedding (without intermittent viral RNA shedding, *n* = 44; with intermittent viral RNA shedding, *n* = 33) are illustrated as scatter plots. Statistical analyses were performed with Mann–Whitney *U*‐test (*P‐value < 0.05). Cytokine level for healthy controls (*n* = 23) is indicated by the black dotted line. Patient samples that are not detectable are presented as the value of logarithm transformation of limit of quantification (LOQ), indicated by the blue dotted line. EGF, epidermal growth factor; FGF‐2, basic fibroblast growth factor; GRO‐α, chemokine (C‐X‐C motif) ligand (CXCL) 1; IL‐17A, interleukin‐17A, IL‐1RA, interleukin‐1 receptor antagonist; IL‐1β, interleukin‐1 beta; IL‐6, interleukin‐6; IP‐10, interferon gamma‐induced protein 10; MCP‐1, monocyte chemoattractant protein 1; PIO, postillness onset; RANTES, regulated on activation, normal T cell expressed and secreted; VEGF‐A, vascular endothelial growth factors A.

Correlations were assessed between levels of immune mediators at median 7 days postillness onset (PIO) (IQR 4–12) and duration of viral RNA shedding in 81 confirmed COVID‐19 patients. The duration of viral RNA shedding was positively correlated with plasma levels of EGF (ρ = 0.29, *P*‐value = 0.008), FGF‐2 (ρ = 0.27, *P*‐value = 0.015), GRO‐α (ρ = 0.31, *P*‐value = 0.005) and RANTES (ρ = 0.30, *P*‐value = 0.006) and inversely correlated with IL‐1β (ρ = −0.25, *P*‐value = 0.025) and IL‐17A (ρ = −0.24, *P*‐value = 0.029). The correlations with epidermal growth factor (EGF), basic fibroblast growth factor (FGF‐2), chemokine (C‐X‐C motif) ligand (CXCL) 1 (GRO‐α) and RANTES(regulated on activation, normal T cell expressed and secreted) were even stronger for patients who required mechanical ventilation (Supplementary table 1). Further stratification of patients based on their duration of viral RNA shedding revealed that patients with prolonged viral RNA shedding (*n* = 41) had different immune profiles at the acute phase compared with patients with viral RNA shedding ≤ 14 days (*n* = 40) (Figure 3a) (Supplementary table 2). Systemic levels of cytokines (EGF, FGF‐2, GRO‐α and RANTES) associated with lung inflammation were significantly higher in patients with prolonged viral RNA shedding, and these patients also demonstrated lower levels of T‐cell cytokines (IL‐17A) at median 7 days PIO (IQR 4–12) (Figure 3a).

To assess whether the immune responses correlated with intermittent viral RNA shedding in confirmed COVID‐19 patients, plasma levels of immune mediators were determined from samples collected at the nearest time point to first PCR negative (median 15 days PIO, IQR 10–19) in patients without intermittent viral RNA shedding (*n* = 44) and compared with samples collected at the nearest time point to first PCR positive (median 16 days PIO, IQR 11–21) during intermittent viral RNA shedding (*n* = 33). There was no significant difference in the sample collection time points between the two groups (*P*‐value = 0.47). The levels of pro‐inflammatory cytokines (IL‐6, IP‐10, MCP‐1 and VEGF‐A) were significantly lower during intermittent viral RNA shedding periods compared with those without intermittent viral RNA shedding pattern (Figure 3b) (Supplementary table 2).

## Discussion

The duration of viral RNA shedding in COVID‐19 ranged from 1 to 35 days. The factors that correlated significantly with the duration of viral RNA shedding on univariate analysis were the requirement for invasive mechanical ventilation and lopinavir/ritonavir treatment. Age, gender, comorbidities and disease severity were not found to be significantly associated with duration of viral RNA shedding. In the multivariate analysis, none of the factors analysed was statistically significant (Table 3). The duration of viral RNA shedding is similar in SARS and MERS coronavirus infections. In SARS, patients with severe disease had higher viral load and 47% of patients with SARS‐CoV had detectable virus RNA at day 21 of illness.^11^ In Middle East respiratory syndrome coronavirus (MERS‐CoV), patients with more severe disease had higher and more prolonged levels of viral RNA tested by RT‐PCR.^12^ The median time to negative test was 17 days in MERS.^13^ As the period of infectivity of COVID‐19 was unknown, the demonstration of 2 consecutive negative RT‐PCR tests was recommended to de‐isolate patients with COVID‐19, similar to MERS.^14^

**Table 3:** Multivariate analysis of factors associated with duration of SARS‐CoV‐2 viral RNA detection

Variable	Multivariable analysis		
	Adjusted odd ratio	95% CI	*P*
Age	1.003	0.98–1.02	0.770
Gender	1.201	0.67–2.17	0.544
Invasive mechanical ventilation	3.073	0.24–17.4	0.204
Lopinavir/ritonavir	1.801	0.68–4.77	0.237

Xu *et al*.^10^ conducted a retrospective study of 113 patients with COVID‐19 and found that male gender, older age, severe disease, late presentation, use of corticosteroids and invasive mechanical ventilation were associated with prolonged viral RNA shedding. Age, gender distribution, comorbidities and days to hospital presentation were similar to our cohort. However, more patients in this study required invasive mechanical ventilation (15.9% vs 5.0%), which could partially explain the longer median viral RNA shedding duration of 17 days. Additionally, 56.6% patients in the cohort received systemic glucocorticoid therapy compared with 0.5% (1 of 201) in our cohort, which has been associated with longer viral RNA shedding in patients with SARS and MERS.^15^, ^16^ In another study of 147 patients with COVID‐19, the use of systemic corticosteroids, but not supplemental oxygen requirement, was associated with prolonged viral RNA shedding.^17^ In contrast, we found on univariate analysis that patients who required invasive mechanical ventilation and patients who received lopinavir/ritonavir treatment to be significantly associated with longer viral RNA shedding. The association of prolonged viral RNA shedding with invasive mechanical ventilation could be confounded by the testing of endotracheal samples which are known to have higher viral load and sensitivity in patients with COVID‐19 pneumonia.^18^ Further, invasive mechanical ventilation was not found to be an independent risk factor of prolonged viral RNA shedding in our multivariable model.

We observed that prolonged viral RNA shedding was associated with elevated levels of cytokines actively involved in pulmonary inflammatory response,^19^, ^20^, ^21^, ^22^ particularly in patients who required mechanical ventilation. Interestingly, patients with prolonged viral RNA shedding demonstrated lower levels of IL‐1β and IL‐17A during the acute phase of infection. Both IL‐1β and IL‐17A are pivotal cytokines involved in activation of anti‐viral T‐cell responses.^23^, ^24^ Exhaustion of T‐cell activation was reported to be associated with increased duration of viral RNA shedding in SARS‐CoV infections.^25^ Hence, we postulate that more effective T‐cell activation at the early phase of infection promotes virus clearance and subsequently shortens viral RNA shedding duration in COVID‐19. Further studies are warranted to comprehensively assess the roles of effector T cells in mediating virus clearance during SARS‐CoV‐2 infection.

Recovered COVID‐19 patients who were diagnosed with re‐infection by subsequent positive RT‐PCR have been reported.^26^ As a result of RT‐PCR test characteristic, very low levels of RNA at the threshold of detection may cause false‐negative or false‐positive results.^27^ This can account for intermittent viral RNA shedding observed in our study. In our cohort, intermittent viral RNA shedding was detected in 77 (38.3%). While most patients had short duration of intermittent viral RNA shedding, 12 (6.0%) had intermittent viral RNA shedding for more than 7 days. In another report, 4 patients who demonstrated 2 negative RT‐PCR tests re‐tested positive again after 5–13 days despite being asymptomatic with no new radiographic changes.^28^ While re‐infection is a concern, prolonged intermittent viral RNA shedding may account for re‐detection of viral RNA.

Viral RNA shedding may be intermittent and is affected by the immune status of the patients. Previous studies have reported that virus re‐activation may be provoked by anti‐inflammatory therapies.^29^, ^30^ We noted that the patients had significantly lower pro‐inflammatory cytokine levels during intermittent viral RNA shedding period, compared with those without intermittent viral RNA shedding after complete virus clearance. A weaker inflammatory response or suppression of inflammatory responses could be triggering virus re‐activation, resulting in intermittent detection in the patients. Whether milder inflammation and virus re‐activation are causally related remains to be explored.

While current de‐isolation strategies generally rely on demonstration of non‐detectable viral RNA, detection by RT‐PCR is only a surrogate marker for infectivity. Positive RT‐PCR test may represent non‐viable viruses or remnant nucleic acid products. A study of 9 patients showed that SARS‐CoV‐2 was readily isolated from respiratory samples within the first week of symptoms, with greater success from sputum than from upper respiratory swabs, and from samples with higher viral loads. Additionally, viral isolation was unsuccessful when patients were beyond day 8 of illness. The authors suggested that patients beyond 10 days of illness with < 100 000 viral RNA copies per mL of sputum could be safely isolated at home.^31^ If the suggested de‐isolation strategy can be applied, it can significantly shorten duration of isolation and demand for scarce hospital beds during periods of peak COVID‐19 activity.

There are limitations to our study. As viral culture was not done, we were unable to determine whether the detection of viral RNA by RT‐PCR was related to viable virus or shedding of remnant non‐viable genetic material. This would have public health implications on the period of infectivity and duration of isolation of COVID‐19 cases. The duration of viral RNA shedding relied on negative tests by RT‐PCR which may be influenced by collection technique or the respiratory site from which the sample was obtained. Patients with predominantly lower respiratory infection may have a negative nasopharyngeal test result. The failure to find any clinical correlates of prolonged viral shedding may have been due to inadequate power to detect small differences, including the small number of patients who required invasive mechanical ventilation.

In conclusion, our study observed no independent risk factor associated with prolonged viral RNA shedding. While the duration of viral RNA shedding varied widely, there were no identified demographic or clinical determinants in the duration of viral RNA shedding. COVID‐19 patients with less active T‐cell responses during the initial phase of infection shed viral RNA longer, and these patients also presented lower levels of pro‐inflammatory cytokines during intermittent viral RNA shedding. Intermittent viral RNA shedding is a common phenomenon, and patients with prolonged intermittent viral RNA shedding may explain reports of re‐detection of viral RNA in recovered COVID‐19 patients.

## Methods

### Patients, clinical data and management

During the study period, all patients with confirmed COVID‐19 infection in Singapore were mandated to be admitted to hospital regardless of illness severity. A total of 201 patients with confirmed COVID‐19 infection admitted in National Centre for Infectious Diseases (NCID) from 22 January 2020 to 5 April 2020 and discharged after obtaining two consecutive negative RT‐PCR results at least 24 h apart were included. The discharge date was censored on 21 April 2020.

Some of the patients received supportive therapy which included supplemental oxygen, empirical antibiotics and/or oral oseltamivir if there was clinical concern of community‐acquired pneumonia (Table 1). Complete blood cell count, renal and liver function, C‐reactive protein (CRP) and lactate dehydrogenase (LDH), and chest radiograph were performed for all patients as standard of care.

Co‐formulated lopinavir–ritonavir, interferon beta‐1b and hydroxychloroquine were prescribed to selected patients at the treating physicians' discretion after shared decision‐making and provision of oral informed consent. Remdesivir was prescribed for patients enrolled in clinical trials in accordance with trial protocols (ClinicalTrials.gov: NCT04280705, NCT04292899, NCT04292730). Corticosteroids were only given for septic shock in the intensive care unit. Demographic, clinical and laboratory data were obtained from the electronic medical records. Data collection was approved under the Infectious Diseases Act with waiver of informed consent.^32^

Respiratory samples for SARS‐CoV‐2 testing included nasopharyngeal swab, throat swab, sputum and endotracheal aspirate. The diagnosis of COVID‐19 infection was confirmed through reverse transcription‐polymerase chain reaction (RT‐PCR) testing for SARS‐CoV‐2. Repeat samples were performed on average, every other day after the patients were afebrile and clinically improving. A cycle threshold value (Ct‐value) above 35 indicates low viral load in the sample; this guided the physicians to repeat viral RNA sampling by PCR 24 h later. National Public Health Laboratory developed test targeting the *N* and *ORF1ab* genes was used at the start of the outbreak in Singapore in late January 2020. From 6 February 2020, a commercial assay was used.^33^ Specific consent for SARS‐CoV‐2 testing was not obtained as all testing was part of routine clinical care. Written informed consent was obtained (approval from the National Healthcare Group Domain Specific Review Board, Study Reference 2012/00917) for measurement of immune mediator serum samples.

Duration of viral RNA shedding was defined as the number of days from symptom onset to the last day of positive RT‐PCR. Duration of persistent viral RNA shedding was defined as the number of days from symptom onset to the first negative RT‐PCR. Duration of intermittent viral RNA shedding, if present, is defined as the number of days from the first negative RT‐PCR test to the last positive RT‐PCR.

### Multiplex microbead‐based immunoassay

Plasma fractions were extracted from blood samples collected from COVID‐19 patients during hospitalisation. Subsequently, plasma from COVID‐19 patients and healthy controls was treated with Triton™ X‐100 solvent/detergent mix (Thermo Fisher Scientific Waltham, MA USA) for virus inactivation (20 min, RT).^34^

Immune mediator levels in inactivated plasma samples were measured with Cytokine/Chemokine/Growth Factor 45‐plex Human ProcartaPlexTM Panel 1 (Thermo Fisher Scientific Waltham, MA USA, #EPX450‐12171‐901), according to the manufacturer's protocol. Kit analyte detection panel included granulocyte–macrophage colony‐stimulating factor (GM‐CSF), epidermal growth factor (EGF), brain‐derived neurotropic factor, beta‐nerve growth factor (bNGF), basic fibroblast growth factor (FGF‐2), hepatocyte growth factor (HGF), monocyte chemoattractant protein (MCP) 1, macrophage inflammatory protein (MIP) 1α, MIP‐1β, RANTES (regulated on activation, normal T cell expressed and secreted), chemokine (C‐X‐C motif) ligand (CXCL) 1 (GRO‐α), stromal cell‐derived factor 1 (SDF‐1α), interferon (IFN) gamma‐induced protein 10 (IP‐10), eotaxin, IFN‐α, IFN‐γ, interleukin (IL) IL‐1α, IL‐1β, IL‐1RA, IL‐2, IL‐4, IL‐5, IL‐6, IL‐7, IL‐8, IL‐9, IL‐10, IL‐12p70, IL‐13, IL‐15, IL‐17A, IL‐18, IL‐21, IL‐22, IL‐23, IL‐27, IL‐31, leukaemia inhibitory factor (LIF), stem cell factor (SCF), tumour necrosis factor (TNF‐α), TNF‐β, vascular endothelial growth factors A and D (VEGF‐A, VEGF‐D), platelet‐derived growth factor (PDGF‐BB) and placental growth factor (PLGF‐1). Samples were obtained from the FLEXMAP^®^ 3D (Luminex) using xPONENT^®^ 4.0 (Luminex) and analysed on Bio‐Plex Manager™ 6.1.1 (Bio‐Rad Hercules, California USA). Standard curves were generated with a 5‐parameter logistic algorithm, reporting values for both mean fluorescent intensity and concentration data. Readout from internal control samples was used to remove any potential plate effects and to obtain a correction factor for normalising assayed plates. The concentrations were logarithmically transformed to ensure normality. Sample with concentration out of measurement range is assigned the value of logarithmic transformation of limit of quantification (LOQ).

### Statistical analysis

The Mann–Whitney *U*‐test was used for comparison of continuous variables, and chi‐square (χ^2^) or Fisher's exact tests for categorical variables as appropriate. Kaplan–Meier survival curve analysis was used to assess the effect of gender, age, severity of illness and experimental anti‐viral agents on the duration of viral RNA shedding. Significant risk factors identified on univariate analysis were further analysed by adjusted multivariate logistic regression analysis to identify independent risk factors associated with the prolonged duration of viral RNA shedding. Non‐parametric Mann–Whitney *U*‐tests were conducted on the logarithmically transformed concentration of immune mediators between patients with prolonged or intermittent viral RNA shedding and those without. Correlation analysis was carried out using Spearman's rank correlation. *P*‐values < 0.05 were considered statistically significant. Data analysis was performed using SPSS, version 26.0 (IBM Corp., Armonk, NY, USA). Plots were generated using GraphPad Prism version 8 (GraphPad Software, San Diego, CA, USA).

## Conflict of interest

The authors declare no conflict of interest.

## Author contributions

**Pei Hua Lee:** Investigation; Methodology; Writing‐original draft; Writing‐review & editing. **Woo Chiao Tay:** Methodology; Project administration; Resources. **Stephanie Sutjipto:** Investigation; Resources. **Siew‐Wai Fong:** Formal analysis; Investigation; Methodology; Software; Writing‐original draft; Writing‐review & editing. **Sean Wei Xiang Ong:** Investigation; Methodology; Writing‐review & editing. **Wycliff Enli Wei:** Data curation; Resources. **Yi‐Hao Chan:** Formal analysis; Investigation; Methodology. **Li Min Ling:** Conceptualization; Resources. **Barnaby E Young:** Conceptualization; Data curation; Methodology; Resources. **Matthias Paul HS Toh:** Data curation; Resources; Validation. **Laurent Renia:** Conceptualization; Resources; Supervision. **Lisa FP Ng:** Formal analysis; Investigation; Methodology; Supervision; Validation; Writing‐review & editing. **Yee‐Sin Leo:** Conceptualization; Visualization. **David C Lye:** Conceptualization; Data curation; Supervision. **Tau Hong Lee:** Conceptualization; Data curation; Formal analysis; Supervision; Validation; Writing‐original draft; Writing‐review & editing.

## Supporting information

Supplementary table 1

Supplementary table 2
