# Supplementary material for: Associations of viral ribonucleic acid (RNA) shedding patterns with clinical illness and immune responses in Severe Acute Respiratory Syndrome Coronavirus 2 (SARS‐CoV‐2) infection
Source: Clin Transl Immunology. 2020 Jul 27;9(7):e1160. doi: 10.1002/cti2.1160 (PMC7385430; doi:10.1002/cti2.1160)
Supplement: Supplementary file 1 — Supplementary table 1 [file CTI2-9-e1160-s001.pdf]

Supplementary Table 1: Correlations between plasma cytokine levels with days of virus clearance in COVID-19 patients

Immune mediator	Correlation with days of virus clearance					
	**Total patients** (n = 81)		**Do not require mechanical ventilator** (n = 62)		**Require mechanical ventilator** (n = 19)	
	rho	*p*	*rho*	*p*	*rho*	*p*
**EGF**	0.2916	**0.0083	0.1888	0.1417	0.5655	*0.0116
**FGF-2**	0.2701	*0.0147	0.1400	0.2779	0.6146	**0.0051
**GRO-α**	0.3126	**0.0045	0.1992	0.1206	0.5775	**0.0096
**RANTES**	0.3015	**0.0062	0.2263	0.0770	0.5176	*0.0232
**IL-1β**	-0.2483	*0.0254	-0.3314	**0.0085	0.0779	0.7511
**IL-17A**	-0.2435	*0.0285	-0.3333	**0.0081	0.0683	0.7812

*Correlation analysis was carried out using Spearman's rank correlation. *p < 0.05, **p < 0.01. EGF, epidermal growth factor; FGF-2, basic fibroblast growth factor; GRO-α, chemokine (C-X-C motif) ligand (CXCL) 1, RANTES, regulated on activation, normal T cell expressed and secreted IL-1β, interleukin-1 beta; IL-17A, interleukin-17A.*
