# Supplementary material for: Associations of viral ribonucleic acid (RNA) shedding patterns with clinical illness and immune responses in Severe Acute Respiratory Syndrome Coronavirus 2 (SARS‐CoV‐2) infection
Source: Clin Transl Immunology. 2020 Jul 27;9(7):e1160. doi: 10.1002/cti2.1160 (PMC7385430; doi:10.1002/cti2.1160)
Supplement: Supplementary file 2 — Supplementary table 2 [file CTI2-9-e1160-s002.pdf]

Supplementary Table 2: Concentrations of immune mediators in COVID-19 patients

No	Immune mediator	Duration of viral RNA shedding from symptom onset Concentration, pg ml^{-1}				Intermittent viral RNA shedding Concentration, pg ml^{-1}			
		≤14 days (n=40)		>14 days (n=41)		Without (n=44)		With (n=33)	
		Median	IQR	Median	IQR	Median	IQR	Median	IQR
**1**	BDNF	24.42	14.04-39.23	32.94	15.80-50.32	29.21	17.61-49.00	30.81	13.56-55.96
**2**	EGF	0.17	0.17-4.28	3.15	0.17-8.67	0.17	0.17-4.41	1.60	0.17-8.24
**3**	FGF-2	0.18	0.18-0.18	0.18	0.18-2.24	0.18	0.18-0.18	0.18	0.18-4.26
**4**	GM-CSF	0.82	0.82-0.82	0.82	0.82-0.82	0.82	0.82-0.82	0.82	0.82-0.82
**5**	HGF	104.40	55.19-170	73.26	45.78-163.00	106.90	67.20-241.60	92.16	41.34-159.40
**6**	PDGF-BB	35.21	18.48-84.48	57.65	20.81-120.40	52.10	21.95-132.10	53.01	20.68-147.20
**7**	PIGF-1	8.60	0.02-25.79	7.85	0.19-32.12	4.11	0.02-23.54	7.94	0.02-43.63
**8**	SCF	4.46	2.78-5.64	4.29	2.57-6.50	3.77	2.74-5.73	4.21	3.10-7.10
**9**	VEGF-A	69.88	45.62-114.80	71.46	32.92-126.30	107.80	43.08-232.30	77.90	30.210-134.80
**10**	VEGF-D	0.18	0.18-0.18	0.18	0.18-6.87	0.10	0.10-3.85	0.10	0.10-0.30
**11**	bNGF	0.87	0.02-12.96	5.83	0.02-18.82	0.74	0.01-14.80	3.46	0.01-21.27
**12**	Eotaxin	15.20	10.17-23.05	16.27	11.42-24.07	15.74	11.05-20.26	15.03	9.85-23.46
**13**	GRO-α	0.23	0.23-0.23	0.23	0.23-3.70	0.05	0.05-0.05	0.05	0.05-1.32
**14**	IP-10	26.89	16.56-42.31	22.95	15.26-57.31	19.11	13.13-35.61	14.76	9.78-20.57
**15**	LIF	4.64	2.80-7.08	4.62	2.95-8.38	5.92	3.70-8.93	5.51	3.41-8.50
**16**	MCP-1	58.00	38.22-101.10	55.07	30.68-96.05	54.90	35.48-104.20	37.06	21.84-53.10
**17**	MIP-1α	2.07	0.11-6.41	2.58	0.11-7.63	3.08	0.11-7.01	2.05	0.11-8.94
**18**	MIP-1β	21.98	5.80-41.80	25.33	3.84-52.95	35.32	19.95-57.12	16.50	4.28-43.61
**19**	RANTES	44.54	29.11-65.82	63.86	32.00-84.12	45.02	28.07-66.94	65.09	28.93-84.71
**20**	SDF-1α	596.30	505.00-695.00	604.6	429.30-687.90	673.90	507.00-785.60	569.40	454.80-684.10
**21**	TNF-α	6.32	1.86-10.39	7.23	2.09-12.94	6.25	1.95-12.45	7.12	0.26-10.53
**22**	TNF-β	149.80	149.80-149.80	149.80	149.80-149.80	2.98	2.98-2.98	2.98	2.98-2.98
**23**	IFN-α	0.02	0.02-0.52	0.02	0.02-4.19	0.02	0.02-0.02	0.02	0.02-0.02
**24**	IFN-γ	13.92	5.41-35.65	25.72	5.77-63.56	8.22	4.44-22.79	10.98	3.61-30.37

**25**	IL-1α	0.01	0.01-0.31	0.01	0.01-0.76	0.02	0.01-1.28	0.01	0.01-0.45
**26**	IL-1β	2.03	1.05-3.60	1.54	0.68-2.82	2.63	1.13-5.12	1.86	0.75-2.49
**27**	IL-1RA	310.80	83.11-600.90	376.70	9.84-972.40	467.50	125.4-1116.00	135.90	20.75-500.70
**28**	IL-2	14.63	5.64-24.19	15.35	3.07-30.65	17.79	7.48-27.36	17.94	1.91-24.70
**29**	IL-4	0.39	0.39-0.39	0.39	0.39-0.39	0.21	0.21-0.21	0.21	0.21-0.21
**30**	IL-5	0.04	0.04-3.01	0.04	0.04-7.70	0.04	0.04-0.16	0.04	0.04-0.04
**31**	IL-6	5.97	0.06-13.27	0.06	0.06-18.49	3.75	0.06-12.55	0.06	0.06-1.78
**32**	IL-7	0.53	0.02-1.22	0.33	0.02-0.94	0.80	0.23-1.77	0.27	0.02-0.62
**33**	IL-8	0.30	0.3-0.3	0.30	0.3-0.3	0.15	0.15-0.15	0.15	0.15-0.15
**34**	IL-9	2.35	2.35-2.35	2.35	2.35-2.35	2.35	2.35-2.35	2.35	2.35-2.35
**35**	IL-10	0.04	0.04-0.04	0.04	0.04-0.04	0.04	0.04-0.46	0.04	0.04-0.04
**36**	IL-12p70	0.63	0.02-1.188	0.95	0.02-2.34	0.39	0.02-1.32	0.98	0.02-2.01
**37**	IL-13	0.20	0.2-4.08	0.2	0.2-0.2	0.20	0.2-2.23	0.20	0.20-0.20
**38**	IL-15	1.07	1.07-17.45	1.07	1.07-11.39	9.35	1.07-22.74	1.07	1.07-17.98
**39**	IL-17A	0.08	0.08-4.075	0.08	0.08-0.08	2.25	0.08-6.17	0.08	0.08-0.21
**40**	IL-18	43.14	27.04-75.31	55.77	21.69-87.38	45.03	23.32-62.97	34.72	17.70-50.97
**41**	IL-21	0.38	0.38-0.38	0.38	0.38-0.38	0.38	0.38-0.38	0.38	0.38-0.38
**42**	IL-22	0.44	0.44-0.44	0.44	0.44-29.45	0.36	0.36-50.80	0.36	0.36-11.94
**43**	IL-23	0.32	0.32-0.32	0.32	0.32-0.32	0.32	0.32-0.32	0.32	0.32-0.32
**44**	IL-27	1.64	1.64-31.03	1.64	1.64-1.64	0.99	0.99-19.66	0.99	0.99-8.50
**45**	IL-31	2.69	2.69-2.69	2.69	2.69-2.69	2.69	2.69-2.69	2.69	2.69-2.69

Interquartile range (IQR); granulocyte-macrophage colony-stimulating factor (GM-CSF); epidermal growth factor (EGF); brain-derived neurotropic factor (BDNF); beta-nerve growth factor (bNGF); basic fibroblast growth factor (FGF-2); hepatocyte growth factor (HGF); monocyte chemoattractant protein (MCP) 1; macrophage inflammatory protein (MIP) 1α, MIP-1β; RANTES (regulated on activation, normal T cell expressed and secreted); chemokine (C-X-C motif) ligand (CXCL) 1 (GRO-α); stromal cell-derived factor 1 (SDF-1α); interferon (IFN) gamma-induced protein 10 (IP-10); interferon alpha (IFN-α), interferon gamma (IFN-γ), interleukin (IL) IL-1α, IL-1β, IL-2, IL-4, IL-5, IL-6, IL-7, IL-8, IL-9, IL-10, IL-12p70, IL-13, IL-15, IL-17A, IL-18, IL-21, IL-22, IL-23, IL-27, IL-31; interleukin-1 receptor antagonist (IL-1RA); leukemia inhibitory factor (LIF); stem cell factor (SCF); tumor necrosis factor-alpha (TNF-α); tumor necrosis factor-beta (TNF-β); vascular endothelial growth factors A and D (VEGF-A, VEGF-D); platelet derived growth factor (PDGF-BB); and placental growth factor (PLGF-1).
